# The effect of educational program based on beliefs, subjective norms and perceived behavior control on doing pap-smear test in sample of Iranian women

**DOI:** 10.1186/s12905-021-01419-w

**Published:** 2021-08-06

**Authors:** Ali Khani Jeihooni, Hanieh Jormand, Pooyan Afzali Harsini

**Affiliations:** 1 Nutrition Research Center, Department of Public Health, School of Health , Shiraz University of Medical Sciences, Shiraz, Iran; 2 Department of Health Education and Promotion, School of Health, Hamadan University of Medical Sciences, Hamadan, Iran; 3 Department of Public Health, School of Health, Kermanshah University of Medical Sciences, Kermanshah, Iran

**Keywords:** HBM model, Behavior control, Iranian women, Pap-smear, Theory of planned behaviour (TPB)

## Abstract

**Objective:**

Cervical cancer is considered treatable as long as it is detected early and managed effectively. Pap smear test is a screening tool that plays an important role in the early detection, prevention and can prevent any early cervical cell changes from becoming cancer. This study aims to survey the effect of educational programs based on beliefs, subjective norms, and perceived behavior control on doing the pap-smear test in a sample of Iranian women.

**Materials and methods:**

This experimental interventional study was performed on 300 women admitted to Fasa City, Fars Province, Iran health centers in 2018–2019. A questionnaire consisting of demographic information, knowledge, Health Belief Model, and Theory of Planned Behavior constructs were used to measure on doing of Pap smear test in women before and after 6 months’ educational intervention.

**Results:**

The results revealed that 6 months after the intervention, 108 women (72%) in the experimental group and only 9 women (6%) in the control group received the Pap smear test.

**Conclusions:**

The current research results revealed that education based on the combination of the health Belief model and theory of planned behavior might be promoting participation and an increasing rate of receiving Pap smear tests in women.

**Supplementary Information:**

The online version contains supplementary material available at 10.1186/s12905-021-01419-w.

## Introduction

Cervical cancer is the sixth most common cancer and is the second principal reason for cancer morbidity in women [1]. Nearly 1.6% of deaths are caused by cancer in women and 15% of deaths are caused by gynecologic cancer [2] so that 440,000 new cases of cervical cancer are reported annually and nearly 80% of them occur in underdeveloped and developing countries [1]. The global prevalence of cervical cancer is estimated to be 11.7%, with its highest prevalence has been reported in the African continent (24%), Eastern Europe (21.4%), and Latin America (16.1%) [1]. Evidence suggests that an increasing number of people are affected by this disease at a global level, especially in Iran [3]. Cervical cancer is the third most common cancer in Iran. Based on the national report of cancer cases registry, the rate of cervical cancer among Iranian women in different provinces has been reported to be 7.1%. Studies suggest that nearly 10% of all invasive cancers in women occur in the uterus, 30% of them occur in the cervix [4]. Nearly one million women are affected by advanced cervical cancer annually, which more than 50% of them die, as globally cervical cancer kills approximately 300,000 women and affects nearly 600,000 women yearly, particularly middle-aged women and those living in lower-resource settings [5]. Cervical invasive cancer is considered preventable cancer through HPV vaccination only which is treatable as long as it is detected early and managed effectively [6], and its 5-year survival rate in the pre-invasive stage is nearly 100% [7]. Doing a regular screening program in this pre-invasive period is highly valuable and can save an individual from the terrible nightmare of cancer [8–10]. Moreover, some studies have reported several causes for cervical cancer, including prolonged uses of oral contraceptive pills, low age at first sexual intercourse, low age at first birth, great number of sexual partners during life, high pregnancy rates, high parity, smoking, illiteracy, low literacy, low economic and social status, among others [11]. Pap smear test is a simple, cost-effective, and uncomplicated method, preferred for screening of cervical cancer [8, 12, 13]. Evidence in several countries suggests that screening programs can reduce cervical cancer mortality rates by up to 60% [14]. However, a large number of women do not, unfortunately, receive a Pap smear test for different reasons, which the most important of them is the fear of having cervical cancer, the painful nature of this test, and shame of doing it, inadequate education, cultural problems, false understandings of the disease, and other psychosocial causes or demographic characteristics [15, 16]. However, screening is an important secondary prevention strategy as cervical cancer is treatable as long as it is detected early [17]. Studies suggest that the rate of Iranian women, who received this test at least once, approximately 28–30%, which is estimated at a low rate [18, 19]. The recommendation on the time of starting for screening and its duration varies in various countries, and there are still controversial views on this issue, depending on the prevalence of the disease in different countries and the cost-effectiveness of the screening method [20]. Based on the Ministry of Health and Medical Education Policies in Iran, the Pap smear test is done in health centers, for women at age group of 20–65 years once per year, and if the result is negative, once per 3 years, based on the conditions specified in the national guidelines [21]. Providing education on promote participation and increasing the rate of receiving Pap smear test is one of the most effective actions taken for early diagnosis of uterine cancer [22]. Though cervical cancer is highly preventable by screening while, hopefully most of cervical invasive cancers are diagnosed in women, who have not received the Pap smear test [16].

As it is mentioned, the rate of receiving Pap smear test in a large number of Iranian women is low, in this regard, the evidence demonstrated intervention education and promotion programs with targeting the developing and promoting motivation factors, could help to promote participation and increasing the rate of receiving Pap smear test in women [9]. The effects of educational interventions greatly depend on using the appropriate models during the selection of educational programs and health problems [23]. Selecting an appropriate health education model or theory is considered as the first step in the planning process of an educational program, and effective health education depends on skill in the use of the appropriate theories and strategies. In this regard, the Health Belief Model (HBM) emphasizes the point that how individual Beliefs and perceptions of the fear of health problems and the assessment of the benefits and barriers of preventive behavior lead to the adoption of behavior [23, 24]. According to this model, one should believe that she is susceptible to a disease such as cervical cancer (perceived susceptibility), women should understand the level of this risk and the seriousness of its various complications in her life (perceived severity) and consider the suggested behaviors such as Pap smear is useful in reducing the risk or severity of her disease (perceived benefits) so that women can overcome the inhibiting factors such as negative emotions, lack of time, etc. (perceived barriers) [25, 26]. Results of Iranian contextualized confirmed that focusing on the perceived benefit and barrier variables of the Health belief model could promoting adherence in pap smear testing in women, also, the perceived benefit and barrier variables have been considered strong predictors of regular pap smear testing among them [27, 28]. Another theory of health education is Theory of Planned Behavior (TPB), based on this theory the best predictor of behavior is one’s intention to be involved in behavior, this key variable is predicted by two variables of behavior-related attitude (general or positive negative evaluation of behavior) and subjective norms (general perception of social pressure to do) [24]. The success of this theory in explaining the behavior depends on the level to which behavior is voluntarily controlled. People often act based on their perception and perception of what others think they should do, and their intention to accept behavior is potentially affected by people who have a close relationship with them. In this theory, one’s subjective norm is the result of multiplying normative beliefs in the motivation to follow the targeted behavior despite these expectations. Additionally, perceived behavior control is defined as the one’s feeling that how much doing or non-doing a behavior is voluntarily controlled [23, 29].

Given conducting previous cross-sectional studies on the target population and the role of people perceptions and beliefs in doing the Pap smear test, the importance of effective subjective norms such as a partner, physicians, and friends, and people perceived behavior control to encourage and empower them on doing Pap smear test [26, 30]. So, the current research was carried out to evaluate the effect of educational intervention based on the combination of the health Belief model and theory of planned behavior on doing of Pap smear test in women.

## Materials and methods

The current research is an experimental interventional and prospective study, carried out on 300 women admitted to Fasa health centers in 2018–2019. Out of 6 urban health centers in Fasa City, 2 centers were randomly selected. Individuals were selected based on the household medical file number and were invited to participate in the research. After selecting the individuals, they were randomly assigned into either the experimental or control group (each group containing 150 subjects). After explaining the research objectives for participants and obtaining informed consent and ensuring the confidentiality of all information, a pre-test questionnaire was completed by two groups. Inclusion criteria: Non-pregnant married women who have had passed at least 6 months of their marriage and without any history of cancer and hysterectomy. Exclusion criteria: non-willingness and informed consent of the women to participate in the research and absence of more than 2 sessions in the educational program. It is essential to be mentioned women in the experimental group received extensive education on cervical cancer and Pap-smear testing, while women in the control group received no educational intervention.

The sample size was calculated 150 women in the experimental group and 150 women in the control group after conducting a pilot study. The pilot study was conducted on 40 women who met the inclusion criteria and were selected randomly by using the sample size formula and considering the (*P* < 0. 05), the confidence level of 95%, and correlation coefficient of 0.37 and considering the drop out of 10%.

$$n = \left( {\frac{{\left( {Z_{{1 - {\raise0.7ex\hbox{$\alpha $} \!\mathord{\left/ {\vphantom {\alpha 2}}\right.\kern-\nulldelimiterspace} \!\lower0.7ex\hbox{2}}}} + Z_{{1 - \beta }} } \right)}}{{0.5 \times ln \left( {\frac{{1 + r}}{{1 - r}}} \right)}}} \right)^{2} + 3$$

The data collection tool in this research was based on other studies [26, 30]. The first part included demographic information of subjects, including age, number of children, age at the first pregnancy, history of receiving the Pap smear, job, education level, being postmenopausal, and the history of cervical cancer in the family.

The second part included questions on the health Belief model and the theory of planned behavior which are design base on theoretical health education and health promotion and methodology researches in this field [23, 24, 31]. The questionnaire of this part includes (1) Assessment of level of knowledge with 15 questions (with yes and no options and score of 0 and 1), (2) Assessing the perceived susceptibility with 5 questions (in 5-point Likert scale option from totally disagree to completely agree, and score 0–4), (3) Assessing the perceived severity with 5 questions (in 5-point Likert scale from completely disagree to completely agree, and score 0–4), (4) Assessing the level of perceived benefits with 6 questions (in 5-point Likert scale from completely disagree to strongly agree and score 0–4), (5) Assessing the level of perceived barriers with 6 questions (in 5-point Likert scale from completely disagree to strongly agree and score 0–4), (6) Assessing the level of subjective norms encouraging for Pap-smear test with 5 questions (in 5-point Likert scale from completely disagree to strongly agree and score 0–4), (7) Assessing the level of perceived behavior control with 2 questions (in 5-point Likert scale from very low to very high and score 1–5), (8) Assessing the level of behavior intention with 2 questions (in 5-point Likert scale from very low to very high and score 1–5). The questionnaire developed for this study is provided as Additional file 1. The validity and reliability of the tools used in cross-sectional studies, conducted on the target population (women admitted to health centers of Fasa) were confirmed. The content validity was evaluated through the impact index item higher than 0.15 and content validity ratio higher than 0.79. For determining the face validity of tools, a list of arranged items was considered by 35 women with similar demographic, economic, and social characteristics with studied subjects. For determining content validity, the ideas of 12 specialists (out of the research team) in health education and promotion (n = 10) and Gerontologist (n = 2) were used. Based on Lawshe's table, items with a CVR value higher than 0.56 for 12 people were considered acceptable and retained for subsequent analysis. Also, in current research, the calculated values for most of the items were higher than 0.70.

According to the calculated Cronbach’s alpha, the total consistency of research tools was 0.88. The consistency of knowledge was 0.86, perceived susceptibility was 0.87, perceived severity was 0.85, perceived benefits was 0.88, perceived barriers was 0.87, perceived behavioral control was 0.82, subjective norms was 0.87 and the behavioral intention was 0.86.

The educational intervention was conducted by the research team after conducting the pilot studies. These pilot studies were carried out to evaluate the factors affecting the Pap smear test based on the health Belief model and theory of planned behavior in women supported by urban centers of Fasa [26, 30]. The educational program focused on the mentioned structures and the considered educational content was prepared based on the educational manual and the educational PowerPoint and film display. The goal of the educational program was to enhance the knowledge of women on cervical cancer, familiarity with the potential risk factors and its complications and symptoms, perceiving the severity of the disease in the samples, and perceiving the benefits of the Pap smear test, identifying the barriers of screening, individual and social management of factors related to Pap smear test, and providing guidelines to prevent cervical cancer. The educational planning in this research was based on an active learning approach and the participants were actively involved in the educational program during the educational intervention and 4 experts of the department manage to control non-communicable diseases in the Fasa Health Center also contributed to the implementation of the program. The educational program for the experimental group was held in eight 50-min educational sessions, once per week in the form of group discussion, brainstorming, question and answer, film display (on mental Beliefs, the positive and negative outcomes of behavior, on the factors facilitating the behavior and the motivation to follow the influential people and the subjective norms), and PowerPoint in the conference hall of the Health Center of Fasa by using gynecologists and health education experts. A session was also held with the presence of spouses, physicians, and health center staff, and their role as supporters and subjective norms in conducting screening behaviors was emphasized. To change the perceived behavioral control on the factors facilitating the Pap smear test, group discussion was held, solutions to improve the willingness of people to do a Pap smear test were provided in each session. At the end of the educational sessions, an educational booklet and an educational CD were provided for them. At the end of educational sessions, an educational message concerning the importance of cervical cancer prevention and screening behaviors was sent to subjects each week and a telegram group was formed for the exchange of information and thoughts, and a follow-up session was held every month. Women were admitted to Fatemiyeh Clinic of Fasa to receive a Pap smear test. Six months after educational intervention, the questionnaire was completed by two experimental and control groups. After the research completion and to observe the research ethics, an educational session on cervical cancer and a Pap smear test was held for the control group. This research was approved in research design at Fasa University of Medical Sciences and an ethics code was received. Data were analyzed using SPSS 22 software, independent *t*-test, Chi-square, and paired *T*-test and the significance level was considered to be 0.05.

## Results

In this research, 300 women supported by Fasa health centers were examined. The mean age of the women in the experimental group and the control group was 32.18 ± 7.34 years and 33.47 ± 7.13 years, respectively, (*P* = 0.136). The mean of the first gestational age in the experimental group and the control group was 19.22 ± 6.66 and 19.98 ± 6.59, respectively, (*P* = 0.162). The mean number of children in the experimental group and the control group was 2.68 ± 1.23 and 2.77 ± 1.34, respectively, (*P* = 0.278). Independent *t*-test showed no significant difference between the two groups.

Chi-square test showed no significant difference between the experimental and control groups in terms of the variables of educational level (*P* = 0.264), job (*P* = 0.120), history of cervical cancer in the family (*P* = 0.153), history of receiving Pap smear test (*P* = 0.122) and being postmenopausal (*P* = 0.253) (Table 1).

**Table 1:** Demographic characteristics of the subjects studied

Variable	Experimental group N = 150		Control group N = 150		*P*-value
	n	%	n	%	
*Education*					
Illiterate	3	2	2	1.33	0.264
Elementary	15	10	18	12	
Secondary school	57	38	54	36	
High school	62	41.33	60	40	
Academic	13	8.67	16	10.67	
*Job*					
Employed	24	16	27	18	0.120
Housekeeper	126	84	123	82	
*History of cervical cancer in the family*					
Yes	9	6	6	4	0.153
No	141	94	144	96	
*History of receiving Pap smear test*					
Yes	58	38.67	62	41.33	0.122
No	92	61.33	88	58.67	
*Being postmenopausal*					
Yes	12	8	14	9.33	0.253
No	138	92	136	9.67	

The results of this research revealed no significant difference between control and experimental groups in terms of the mean score of knowledge (*p* = 0.09), perceived susceptibility (*p* = 0.104), perceived severity (*p* = 0.135) and perceived benefits (*p* = 0.176), perceived barriers (*p* = 0.121), perceived behavioral control (*p* = 0.289), subjective norms (*p* = 0.332), and behavioral intention (*P* = 0.335), but significant difference was found between them 6 months after educational intervention (*P* < 0.05). A paired *t*-test (*p* = 0.05) showed that the mean score of knowledge, perceived susceptibility, perceived severity, perceived benefits, perceived behavioral control, subjective norms, and behavioral intention were significantly increased in the experimental group and the mean score of perceived barriers decreased (*P* < 0.05). In the control group, the mean score of knowledge, perceived susceptibility, perceived severity, perceived benefits, perceived barriers, perceived behavioral control, subjective norms, and behavioral intention did not change significantly (*P* > 0.05) (Table 2).

**Table 2:** Comparison of the mean scores of knowledge, perceived susceptibility, perceived severity, perceived benefits, perceived barriers, perceived behavioral control, subjective norms, and behavioral intention of the subjects before and 6 months after intervention in the experimental and control groups

Variable	Group	Before intervention	6 months after intervention	Paired *t*-test
Knowledge	Experimental	6.55 ± 2.09	11.20 ± 2.47	0.001
	Control	7.11 ± 2.58	7.26 ± 2.66	0.130
	Independent *t* test	0.09	0.001	
Perceived susceptibility	Experimental	8.32 ± 2.15	15.11 ± 2.64	0.001
	Control	8.40 ± 2.86	9.15 ± 2.78	0.148
	Independent *t* test	0.104	0.001	
Perceived severity	Experimental	8.32 ± 2.12	16.14 ± 2.16	0.001
	Control	7.97 ± 2.86	8.20 ± 2.91	0.172
	Independent *t* test	0.135	0.001	
Perceived benefits	Experimental	8.70 ± 2.43	18.26 ± 2.71	0.001
	Control	9.04 ± 2.36	10.12 ± 2.22	0.198
	Independent *t* test	0.176	0.001	
Perceived barriers	Experimental	18.16 ± 2.52	7.26 ± 2.57	0.001
	Control	18.78 ± 2.35	17.46 ± 2.41	0.256
	Independent *t* test	0.121	0.001	
Behavioral control	Experimental	4.13 ± 1.08	8.34 ± 1.09	0.001
	Control	4.25 ± 1.10	5.68 ± 1.13	0.412
	Independent *t* test	0.289	0.001	
Subjective norms	Experimental	8.12 ± 2.42	16.19 ± 2.28	0.001
	Control	7.90 ± 2.75	8.14 ± 2.76	0.166
	Independent *t* test	0.322	0.001	
Behavioral intention	Experimental	4.06 ± 0.79	8.38 ± 1.13	0.001
	Control	4.14 ± 0.97	4.27 ± 1.11	0.528
	Independent *t* test	0.355	0.001	

The results revealed that 6 months after the intervention, 108 women (72%) in the experimental group and only 9 women (6%) in the control group received the Pap smear test (*P* < 0.05).

## Discussion

In cross-sectional research carried out on 700 women supported by health centers of Fasa city, 45.7% of the participants had a history of receiving Pap smear test, and 20.7% of them received this test, and 6.86% of women had a history of cervical cancer in the family [30].

The results showed that the mean score of knowledge of women in experimental and control groups concerning cervical cancer and its prevention was at a low level, but 6 months after educational intervention, the mean score of knowledge in the experimental group showed a significant increase comparing to control group. The research carried by Donati et al. showed that Italian women who participated in the research had aged 18–26 years in 2008, had a low level of knowledge and false understanding of uterine cancer and its prevention. The subjects considered the health care providers as their most trusted source to receive the medical information [32]. As the population studied in this research included the women supported by health centers, providing educational programs by using educational theories such as theory of planned behavior and the health Belief model by the staff of these centers would be an important action to enhance the knowledge, attitude, and practice of women on the Pap smear test. In a research carried out by Sha-mohammadi et al., they investigated the factors affecting the doing the Pap smear test based on a combination of the theory of planned behavior and the stages of behavior change. The model structures predicted 31% of the behavioral intention variance, and most people were in the pre-thinking stage [33]. In a research carried out by Hazavehei et al. on 124 women in Khomeinishahr city, educational intervention based on the health Belief model increased the knowledge score of women in the experimental group after the educational intervention [34]. In qualitative research carried out by Keshavarz et al. using a combination of theory of planned behavior and self-efficacy on 70 women aged 20–45 years, it was found that the knowledge level and attitude of women toward breast and cervical cancer screening methods were at a low level [35]. The results of studies conducted by Park Somi et al. [36], Twinn et al. [37], Karimy et al. [38], Yakhforoushha et al. [39], and Arshad et al. [40] are in line with those of this research.

The research results showed that the mean score of perceived susceptibility and perceived severity of both groups were low before the research, indicating that women of both groups did not consider themselves at risk for cervical cancer, while in the research conducted on women in Botswana, most of the women considered themselves at risk for cervical cancer [41]. Six months after the educational intervention, the mean score of perceived susceptibility in the experimental group was significantly higher compared to that in the control group. Health educators require taking action to develop perceived susceptibility and perceived severity by describing the probability of incidence of negative outcomes and exposing the risks for the clients. In research carried out by Bahmani et al. on 180 women, the educational intervention increased the mean score of perceived susceptibility in the experimental group, but the mean score of perceived severity did not change [42]. In cross-sectional research carried out by Jeihooni et al., the structures of perceived susceptibility and perceived benefits were the most important predictors of the Pap smear test [26]. In the research carried out by Jowzi et al., no significant relationship was found between doing Pap smear test, perceived vulnerability, and perceived severity [43]. In research, Khademolhosseini et al. evaluated the effect of educational intervention based on the health Belief model through the telegram message on Pap smear performance of women. It was found that knowledge level, susceptibility, and perceived severity of people increased 3 months after the intervention [44]. The results of other studies are also in line with those of this research [34, 39, 45–49].

The research results revealed no difference between the experimental and control groups concerning perceived benefits and perceived barriers before the educational intervention, but educational intervention increased the mean score of perceived benefits and reduced the mean score of perceived barriers in the experimental group, while no significant difference was observed in the control group. In analyzing the behavior of women on Pap smear, a significant relationship was found between the perceived benefits and reduced barriers and performance based on the health Belief model [27]. In this research, education based on the health Belief model resulted in a better perception of the benefits by the experimental group and overcome the barriers more effectively. In their research, McFarland et al. reported that women who had more barriers received less Pap smear over the last 5 years [41]. In the research carried out by Somi Park et al. a significant difference was found between the experimental and control groups after the intervention in cognitive barriers and barriers related to doing the Pap smear. They justified this difference as the effect of educational intervention and providing information on cervical cancer and discussion on the value of Pap smear in educational sessions [36]. In cross-sectional research carried out by Jeihooni et al., the level of susceptibility, severity, perceived benefits, and perceived barriers in subjects who had a history of Pap smear test were higher compared to those who did not have this test. The most common reason for the test was health care staff guidelines, and the most important reason for the non-test was the Belief that they are not prone to cervical cancer [26]. The results of the research carried by Rakhshani et al. [50], Karimy et al. [38], Bahmani et al. [42], and Park et al. [36] are in line with the results of the current research. The mean score of perceived behavioral control in the experimental group was significantly higher than that in the control group 6 months after the intervention. Education on cervical cancer and doing a Pap smear test through educational sessions and providing appropriate incentive and informational feedback in group discussions improved the perceived behavioral control in the experimental group. Perceived behavior control and the feeling that behavior is voluntary is one of the important factors in taking step to perform that behavior [51], and increasing perceived behavioral control following educational intervention indicates the impact of education in developing this perception that “if I want, I can do Pap smear test”. The research carried out by Roncancio et al. revealed that perceived behavioral control and subjective norms are positively correlated with the intention for cervical cancer screening [52]. In the research carried out by Linton, perceived behavioral control showed direct effect on Pap-smear behavior [53].

In the research carried out by Ogilvie et al. perceived behavioral control and subjective norms predicted cervical cancer screening [54]. In the research conducted by Teitelman et al. the perceived behavioral control and subjective norms predicted the intention for Pap smear test [55]. In cross-sectional study research carried out on married women admitted to health centers in Fasa, a significant relationship was found between knowledge, abstract norm, and perceived behavioral control and screening behavior [30]. Consistent with findings of the current research, Sweeney et al. [56], Sha-Mohammadi et al. [33], the educational intervention increased the mean score of perceived behavioral control in the experimental group. The current research findings revealed that the mean score of subjective norms in the experimental group was significantly higher than that in the control group 6 months after the intervention. The subjective norms are among theory of planned behavior structures, referring to effective social pressures for adopting the considered behavior. When the social pressures and supports of the family members, especially spouses, physicians, health care staff, and friends to perform health behaviors by an individual are high, adopting that behavior by that individual would be also high [57]. In the research conducted by Keshavarz et al. decreased level of subjective norms especially support of partner and low knowledge was reported as negative factors for cervical cancer screening [58]. In research carried out on Taiwanese women, subjective norms predicted cervical cancer screening [59]. In research conducted by Jalilian et al., the most important predictor for Pap smear test was found to be subjective norms. In this research, spouse, physician, and educational programs were found to be influential factors [29]. In research carried out on 206 women aged 18–26 years in Singapore, Chrayil et al. found that subjective norms were the most important predictors of the Pap test, and perceived behavior control had the moderate predictive power [60]. The research carried out by Silva et al. revealed that social support had a positive effect on the Pap smear test [61]. In the research conducted by Karimy et al. the most important guide to Pap test was health staff [38]. The results of other studies are consistent with those of the current research [38, 62–64].

This research results revealed that the mean score of behavioral intention in the experimental group increased during the 6 months after the intervention, while no change was seen in the control group. Six months after the intervention, 72% of the experimental group subjects and 6% of control group subjects received the Pap smear, indicating the effect of educational intervention on the Pap test in the experimental group. The intention is the most important structure in this regard so that by changing this structure, more adoption of the self-care behaviors can be expected from people [30]. In the research carried out by Moradi et al. theory of planned behavior predicted 57.4% of the intention variance and 31.6% of Pap smear screening test variance [30]. In the research carried out by Roncancio et al. variance value was 27.6% for intention and 13% for cervix screening [52]. In a research carried out by McClenahan et al. they used a combination of the health Belief model and theory of planned behavior for testicular self-Examination (TSE). The theory of planned behavior predicted 50% of intention variance and 22% of behavior variance and the health Belief model predicted 56% of intention variance and 21% of behavior variance [65].

In the research carried out by Sha-Mohammadi et al. [33], the educational intervention increased the mean score of intention and screening behaviors in the experimental group subjects. In other studies, it was found that educational intervention resulted in Pap smear test screening [42, 44, 46, 66].

The limitations of the present study included the use of self-report method for data collection which limit the generalizability of the results. Regardless of these limitations, this study has some advantages. This study was the direct education approach in educational strategy which the result of our study along with other studies showed advantages of using this strategy compare to other different health education approaches and methods (such as calls, mailed postcards…) [67, 68].

## Conclusion

The current research results revealed that education based on the combination of the health Belief model and theory of planned behavior might be promoting participation and an increasing rate of receiving Pap smear tests in women. The reason for the effectiveness of the program used in this research is recognizing the weaknesses before the intervention and developing educational strategies and content based on them. This research results suggest that when women gain adequate knowledge on cervical cancer and Pap smear test, see themselves as prone to it, take its risk seriously, obtain the high perception of behavioral control to do Pap smear test, feel fewer barriers to perform the behavior, and subjective norms such as support of family member and health care staff cooperate, the intention and performing the behavior in women would also enhance.

Therefore, Well-planned, comprehensive and integrated approach, based on clients' needs and requirements, is the most important characteristic of a successful screening program. It should include health education and health promotion elements, and interventions of the health care staff need to be based on these principles. Also, comprehensive educational programs through mass media such as TV and newspapers would be helpful in this regard. Similar studies are required to use other behavior change models for regular and continuous evaluation of behavior change at longer intervals (more than 6 months).

## Supplementary Information

**Additional file 1**. The questionnaire of this study.

## Data Availability

All supporting data are available through the corresponding author.

## Abbreviations

HBM: Health Belief Model
TPB: Theory of Planned Behavior
