# Supplementary material for: The effect of educational program based on beliefs, subjective norms and perceived behavior control on doing pap-smear test in sample of Iranian women
Source: BMC Womens Health. 2021 Aug 6;21:290. doi: 10.1186/s12905-021-01419-w (PMC8348997; doi:10.1186/s12905-021-01419-w)

|  |
| --- |

**Code of participant:**

**Age:………..**

**First gestational age:……………**

**Number of children:………………**

**Educational level:** Illiterate
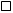
 Elementary
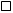
 Secondary School
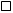
 High School
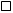
 Academic
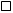


**Job:** Employed
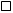
 Housekeeper
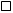


**History of cervical cancer in the family:** Yes
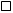
 No
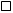


**History of receiving Pap smear test:** Yes
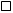
 No
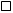


**Being postmenopausal:** Yes
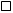
 No
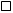


| **No** | **Yes** | **Knowledge** | |
| --- | --- | --- | --- |
|  |  | **Pap smears are the most helpful way to detect pre-cancer and cancer of the cervix** | 1 |
|  |  | **Women should have Pap smears at least every three years.** | 2 |
|  |  | **Pap smear is not able to detect pre-cancerous cells before the manifestation of their symptoms** | 3 |
|  |  | **The purpose of the Pap smear is to detect abnormal cells in the cervix** | 4 |
|  |  | **Pap smear is not successful in reducing the incidence and mortality of cervical cancer.** | 5 |
|  |  | **Pap smears can detect all types of female genital cancer.** | 6 |
|  |  | **Pap smear is a non-invasive and relatively inexpensive method.** | 7 |
|  |  | **Women should have a Pap smear since the onset of sexual activity** | 8 |
|  |  | **In Pap smear, cervical cells are examined.** | 9 |
|  |  | **Pap smears can be performed at both menstrual and non-menstrual periods.** | 10 |
|  |  | **A woman should not have sex 24 hours before having a Pap smear.** | 11 |
|  |  | **Pap smears should be discontinued after menopause.** | 12 |
|  |  | **If someone is having a normal Pap smear, she does not need Pap smears in the future.** | 13 |
|  |  | **There is no need to have a Pap smear if it is not administered by a doctor.** | 14 |
|  |  | **Pelvic pain is one of the first signs of cervical cancer.** | 15 |

| Strongly agreed | Agreed | Neither agree nor disagree | Disagreed | Strongly disagreed | **Perceived Susceptibility** | No |
| --- | --- | --- | --- | --- | --- | --- |
|  |  |  |  |  | **I'm worried about getting cervical cancer.** | 1 |
|  |  |  |  |  | **I'm worried about my friends and acquaintances getting cervical cancer** | 2 |
|  |  |  |  |  | **I am healthy, young, and strong enough not to get cervical cancer.** | 3 |
|  |  |  |  |  | **Because I do not have a vaginal infection, cervical cancer can be my problem.** | 4 |
|  |  |  |  |  | **Because I do not use hormonal contraceptives, cervical cancer cannot be my problem.** | 5 |

| Strongly agreed | Agreed | Neither agree nor disagree | Disagreed | Strongly disagreed | **Perceived Severity** | No |
| --- | --- | --- | --- | --- | --- | --- |
|  |  |  |  |  | **Cervical cancer is one of the most important and common cancers in women.** | 1 |
|  |  |  |  |  | **Cervical cancer can be asymptomatic in the early stages.** | 2 |
|  |  |  |  |  | **Cervical cancer is a treatable disease.** | 3 |
|  |  |  |  |  | **The name cancer causes me fear and panic.** | 4 |
|  |  |  |  |  | **If I get cervical cancer, everything will end for me.** | 5 |

| Strongly agreed | Agreed | Neither agree nor disagree | Disagreed | Strongly disagreed | **Perceived Benefits** | No |
| --- | --- | --- | --- | --- | --- | --- |
|  |  |  |  |  | **Pap smear test causes early detection of cervical cancer and treatment for it.** | 1 |
|  |  |  |  |  | **Pap smear test is very simple.** | 2 |
|  |  |  |  |  | **Pap smear testing ensures that a person is in good health.** | 3 |
|  |  |  |  |  | **Pap smears are easier to treat than cervical cancer.** | 4 |
|  |  |  |  |  | **If cervical cancer is diagnosed early, it can be prevented from progressing to other cancers.** | 5 |
|  |  |  |  |  | **Pap smear is effective in diagnosing cervical cancer.** | 6 |

| Strongly agreed | Agreed | Neither agree nor disagree | Disagreed | Strongly disagreed | **Perceived Barriers** |  |
| --- | --- | --- | --- | --- | --- | --- |
|  |  |  |  |  | **Sampling is difficult for me, and I am generally ashamed and disgusted by any kind of gynecological examination.** | 1 |
|  |  |  |  |  | **It is time-consuming for me to have a Pap smear.** | 2 |
|  |  |  |  |  | **If I have cervical cancer, I prefer to be unaware of it.** | 3 |
|  |  |  |  |  | **Disease and cancer are the results of divine destiny.** | 4 |
|  |  |  |  |  | **I do not have a Pap smear because I am afraid the result will be positive.** | 5 |
|  |  |  |  |  | **I doubt the effectiveness of Pap smears for diagnosing and preventing cervical cancer** | 6 |

| Strongly agreed | Agreed | Neither agree nor disagree | Disagreed | Strongly disagreed | **Subjective Norms** |  |
| --- | --- | --- | --- | --- | --- | --- |
|  |  |  |  |  | **My partner encourages me to have regular Pap smears.** | 1 |
|  |  |  |  |  | **My closest friends think that it is better to have a regular Pap smear.** | 2 |
|  |  |  |  |  | **My doctor thinks it is better to have a regular Pap smear.** | 3 |
|  |  |  |  |  | **Health professionals think it is best to get regular Pap smears.** | 4 |
|  |  |  |  |  | **Training programs encourage me to have regular Pap smears** | 5 |

| very high | high | moderate | low | very low | **Perceived Behavioral Control** |  |
| --- | --- | --- | --- | --- | --- | --- |
|  |  |  |  |  | **I will do a scheduled Pap smear test even if I do not have time.** | 1 |
|  |  |  |  |  | **I perform a scheduled Pap smear even if I am afraid of a positive result.** | 2 |

| very high | high | moderate | low | very low | **Behavioral Intention** |  |
| --- | --- | --- | --- | --- | --- | --- |
|  |  |  |  |  | **I plan to pursue my doctor on how to prepare for a Pap smear test.** | 1 |
|  |  |  |  |  | **I plan to perform a Pap smear test in the next 3 months.** | 2 |

**In 6 months after educational intervention**

**Behavior:** I perform the Pap smear test: Yes
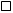
 No
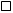

Supplement: Supplementary file 1 — Additional file 1. The questionnaire of this study. [file 12905_2021_1419_MOESM1_ESM.docx]
